# Recurrent hot droughts cause persistent legacy effects in a temperate Scots Pine forest

**DOI:** 10.1111/plb.70066

**Published:** 2025-06-16

**Authors:** S. Haberstroh, A. Christen, M. Sulzer, F. Scarpa, C. Werner

**Affiliations:** ^1^ Ecosystem Physiology, Faculty of Environment and Natural Resources University Freiburg Freiburg Germany; ^2^ Environmental Meteorology, Faculty of Environment and Natural Resources University Freiburg Freiburg Germany

**Keywords:** *Carpinus betulus*, enhanced vegetation index, net ecosystem carbon exchange, *Pinus sylvestris*, sap flow, water potential

## Abstract

- Recent hot‐dry events have caused significant impacts and legacy effects in temperate ecosystems. Here, we investigate legacy effects of the 2018 hot drought on a *Pinus sylvestris* L. forest in southwestern Germany and the effects of post‐2018 recurrent hot‐droughts on ecosystem carbon fluxes.
- We combined ecophysiological, remote sensing (Enhanced Vegetation Index, EVI) and micrometeorological (Net Ecosystem Carbon Exchange, NEE) measurements to assess past and present ecosystem functioning.
- We found strong and persistent legacy effects and high tree mortality of *P. sylvestris*, with deciduous understorey trees slowly replacing *P. sylvestris*. After 2018, EVI clearly followed the pattern of a deciduous‐dominated forest, indicating changes in canopy structure, type and seasonality in NEE. Significant legacy effects in NEE were found and the ecosystem shifted from a carbon sink (NEE = −391 ± 204 g C m^−2^ year^−1^, 2003–2006) to carbon neutral (NEE = +13 ± 28 g C m^−2^ year^−1^) in 2021, a cold and wet year. All other years post‐2018 were hotter and drier than the long‐term average (1991–2020), and the ecosystem was turning into a carbon source, with highest values in 2022 (NEE = +329 ± 19 g C m^−2^ year^−1^). These compound events of atmospheric and edaphic drought led to strong ecosystem carbon release post‐2018.
- Our data show that the ecosystem most likely experienced strong drought legacy effects, such as 2018, at species, community and ecosystem scales. These negative effects were further exacerbated by recurrent atmospheric and edaphic droughts, shifting the ecosystem to a net carbon source after 2018.

## Graphical abstract

A series of hot drought events caused persistent legacy effects in a Scots Pine forest, with severe negative impacts on ecosystem carbon fluxes.

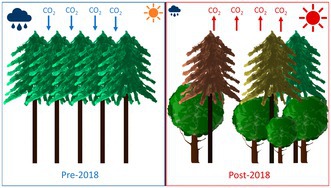

## INTRODUCTION

In recent years, temperate ecosystems have experienced significant droughts (e.g. Schuldt & Ruehr 2022), which can evoke distinct drought responses, for example, reduced carbon (e.g. Van der Woude *et al*. 2023; Scapucci *et al*. 2024) and water (e.g. Haberstroh *et al*. 2021; Knüver *et al*. 2022; Gebhardt *et al*. 2023) fluxes. Plant stress is further enhanced when these periods are accompanied by (untypical) high air temperature and high vapour pressure deficit (VPD) (Grossiord *et al*. 2020). While the immediate response of ecosystems to such abiotic conditions can have substantial impacts on seasonal and annual ecosystem water and carbon budgets, drought legacy effects can have long‐lasting (years to decades to permanent) negative effects on ecosystems (e.g. Anderegg *et al*. 2015; Kannenberg *et al*. 2020). Drought legacy effects are defined as changes or responses of ecosystem properties or processes in the post‐drought phase. These alterations can be positive or negative (Müller & Bahn 2022; Vilhonen *et al*. 2022), however negative legacy effects are more common in forest ecosystems (Anderegg *et al*. 2015; Vilhonen *et al*. 2022). Such drought legacy effects in the temperate forest ecosystems of Central Europe are increasing (e.g. Bastos *et al*. 2020a; Yu *et al*. 2022; Pohl *et al*. 2023) and thus, in the focus of current scientific research. However, there is still debate about how to assess drought legacy effects in a common framework (Kannenberg *et al*. 2020), especially because they occur at different spatial and temporal scales and interact with other abiotic and biotic stressors (Kannenberg *et al*. 2019). Müller & Bahn (2022) suggest assessment of drought legacies (duration and size) in the post‐recovery or post‐drought period, that is, after ecosystem recovery, against a pre‐defined baseline without stress to allow for better comparability between studies.

According to Müller & Bahn (2022), drought legacy effects can occur on three different scales: the species, ecosystem, and community scales, which are all inherently connected and influence each other. Legacy effects at the species scale include, among others, reduced growth rates (e.g. Kannenberg *et al*. 2020) or reduced water fluxes (e.g. Gebhardt *et al*. 2023; Ruehr & Nadal‐Sala 2026) of single trees following drought. At the community scale, shifts in species composition or dominant tree species can alter the whole ecosystem structure (Müller & Bahn 2022). Often, changes in vegetation composition follow drought related tree mortality events and have been observed across multiple biomes globally (Batllori *et al.*, 2020). For example, in a Patagonian forest, an extreme drought event led to a shift in dominance from *Nothofagus dombeyi* towards the drought‐tolerant species *Austrocedrus chilensis* (Suarez & Kitzberger 2008). An experimental drought treatment in a Mediterranean holm oak ecosystem indicated a vegetation shift from *Quercus ilex* to *Phillyrea latifolia* under progressing drought (Liu *et al*. 2018). In the Swiss Rhone valley, *P. sylvestris* forests are developing into *Quercus pubescens* dominated ecosystems as a consequence of drought (Rigling *et al*. 2013). Often, these changes in species composition are permanent (e.g. Hillebrand & Kunze 2020; Müller & Bahn 2022).

Legacy effects at the community scale are often directly interconnected to the ecosystem scale, which includes, among others, ecosystem carbon and water cycling or plant–plant interactions (Müller & Bahn 2022). For example, after the 2018 hot drought, in 2019 net ecosystem productivity was reduced by 93 g C m^−2^ (−25%) in a temperate mixed deciduous forest compared to pre‐drought years (Pohl *et al*. 2023). Effects at the ecosystem scale are highly connected to legacy effects at the species and community scales with possible feedbacks (Müller & Bahn 2022).

The accurate assessment of drought legacy effects at different scales is complicated by the co‐occurrence of other abiotic and biotic stressors, which can affect the different scales in multiple manners. Moreover, interactions of different stressors are complex and difficult to predict, but often lead to a stronger impact than one stressor alone (Zscheischler *et al*. 2018). For example, the occurrence of high VPD during periods of edaphic drought has been linked to reduced photosynthesis and stomatal conductance, but also to an increased risk of plant mortality (Grossiord *et al*. 2020). Such compound events (Zscheischler *et al*. 2018) have and will increase, especially in the combination of hot‐dry events (Bevacqua *et al*. 2022). In Central Europe, 2018 (Schuldt *et al*. 2020; Schuldt & Ruehr 2022) and 2022 (e.g. Copernicus Climate Change Service 2022; Schmied *et al*. 2023; Scapucci *et al*. 2024) can be classified as hot‐dry compound events with devastating effects on ecosystems (e.g. Schuldt *et al*. 2020; Bastos *et al*. 2020b). Compared to the hot drought in 2003, which used to be the benchmark for hot droughts in Europe, 2018 was even more intense and affected an area approx. 1.5‐times the size of 2003 (Buras *et al*. 2020; Bastos *et al*. 2020a). In addition, 2018 was preceded by very dry conditions, starting already in 2014 (Moravec *et al*. 2021), and followed by a series of dry and hot years in Central Europe, such as 2019, 2020 and 2022 (Knutzen *et al*. 2025). Thus, impacts on ecosystems in Central Europe of the 2018 hot drought were more severe than in 2003 (e.g. Buras *et al*. 2020). For example, Thompson *et al*. (2020) found that the temperate forests of Europe reduced their carbon uptake by 90 ± 60 Tg C year^−1^ in 2018 compared to 2009–2018. Likewise, Van der Woude *et al*. (2023) quantified the reduction of carbon uptake by the biosphere to be 56–62 Tg C in the drought affected area of Central and Southeastern Europe in the summer months (June–August) of 2022.

Yet, also 2019, 2020 and 2023 showed above average air temperature and low precipitation in many regions in Central Europe (Copernicus Climate Change Service 2019, 2020, 2023), including extreme events, such as heatwaves and strong soil moisture deficits. Thus, the occurrence of recurrent drought and heat, prior to the complete recovery from the previous extreme event has been a frequent phenomenon in temperate ecosystems of Central Europe in the last decades, which further challenges the correct assessment of drought legacy effects (Müller & Bahn 2022; Werner *et al*. 2024). Only 2021 was wet and cool enough in Central Europe (Climate Change Service 2021) to allow the possibility for proper ecosystem recovery. Thus, an increasing number of ecosystems are at the edge of permanent and persistent damage (e.g. Lloret & Batllori 2021; George *et al*. 2022), some even reaching their tipping point, where a new ecosystem state is establishing due to increased mortality rates of former dominant key species (e.g. Batllori *et al*. 2020; Haberstroh *et al*. 2022).

A species widely distributed over Europe, which is severely suffering from (recurrent) drought events is Scots Pine (*Pinus sylvestris* L.), even in areas of its climate optimum (Bose *et al*. 2024). Recurrent extreme drought events of recent years, in combination with biotic agents, such as mistletoe infection or insect infestation, have led to increased dieback of *P. sylvestris* (Lemaire *et al*. 2022) in several areas of Europe, spanning from Spain to northern Germany (Bose *et al*. 2024). Although long‐considered a drought‐ and frost‐tolerant species with low site requirements (Krakau *et al.*, 2013), the suitability of *P. sylvestris* for (productive) forestry under future climate change is increasingly questioned (Brichta *et al*. 2023; Diers *et al*. 2024; Knocke *et al*. 2024).

A *P. sylvestris* site with a high level of drought impact can be found in SW Germany, in the Upper Rhine valley at the Hartheim Forest Research Site, which has provided continuous climate and tree‐level observations since 1978. The site is an associated ecosystem monitoring site of the Integrated Carbon Observation System (ICOS, Site code DE‐Har, Heiskanen *et al*. 2022). Even before 2018, there were signs of drought stress in this ecosystem. However, during the 2018 hot drought, midday needle water potential (*Ψ*) of *P. sylvestris* trees dropped to −7.5 MPa, triggering xylem cavitation and embolism formation. Consequently, roughly half of *P. sylvestris* trees at DE‐Har had died up to September 2020, and the ecosystem shifted to a carbon source in 2019 (Haberstroh *et al*. 2022). Thus, this ecosystem is ideally suited to investigate drought legacy effects, but also the impact of recurrent drought and heat events of recent years.

The main objectives of this study are (1) to identify persistent legacy effects in this drought‐prone *P. sylvestris* forest at different scales, as well as potential feedbacks between scales, and (2) to investigate the impact of recent hot drought events on critical ecosystem processes, such as ecosystem carbon fluxes. We combine measurements at the species (radial sap flow and water potential 5 years after 2018), community (vegetation dominance as depicted by the enhanced vegetation index) and ecosystem level (net ecosystem carbon exchange, NEE) to elucidate the impact of the 2018 hot drought and subsequent compound events until 2023.

We hypothesize that (a) the ecosystem, but especially *P. sylvestris*, has severely suffered from drought legacy effects of the 2018 hot drought at all scales, and that this may induce a shift in species dominance in favour of deciduous species. Further, (b) we postulate that co‐occurring atmospheric and edaphic droughts (i.e. *compound droughts*) since 2018 in combination with persistent drought legacy effects may shift the ecosystem to an annual net carbon source.

## MATERIALS AND METHODS

### Experimental site and design

**Fig. 1:**
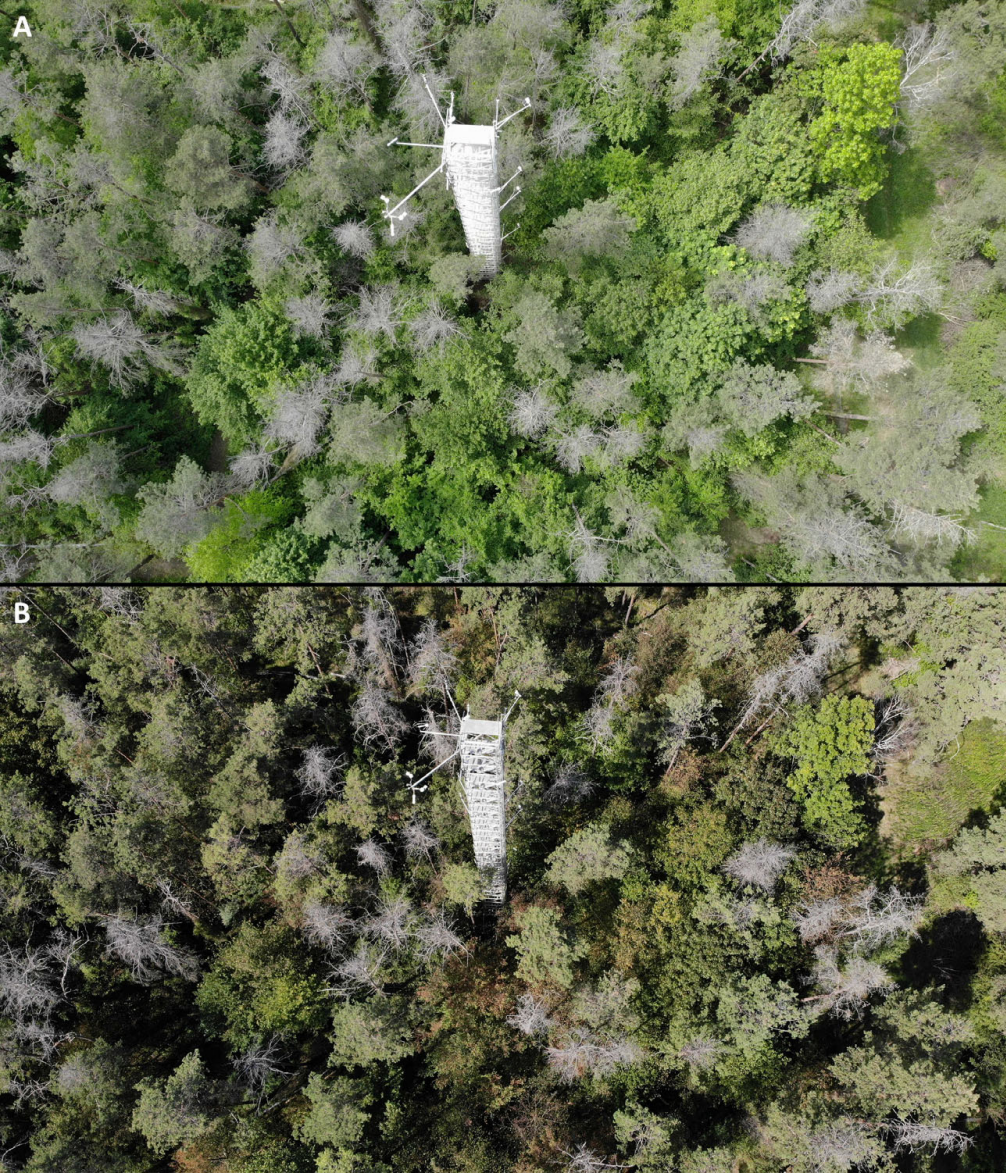
Aerial view of the *Pinus sylvestris* ecosystem with the large canopy access tower (30 m) in May (A) and September (B) 2023. Dead *P. sylvestris* trees, gaps in the forest canopy and a dense layer of understorey trees are visible throughout the forest.

The study was carried out at the permanent Hartheim Forest Research Site DE‐Har (7.59814° E, 47.93391° N, 201 m a.s.l.). DE‐Har is located in the Upper Rhine Valley, SW Germany. The field site was established in 1974 and is located in a slow‐growing *Pinus sylvestris* L. forest planted in 1963 (Holst *et al*. 2008). The mean annual air temperature (*T*_*air*_) is 10.6°C and the mean annual precipitation is 646 mm (1991–2020). In the growing season (April–September), mean *T*_*air*_ is 16.1°C, with a mean precipitation of 391 mm (1991–2020) (Christen *et al*. 2024a). Access to the canopy is currently provided by two towers (18 and 30 m; Fig. 1 and Fig. S1), the taller tower provides continuous measurements of above‐canopy radiation fluxes, sensible heat, water and CO_2_ fluxes by eddy covariance as part of ICOS.

The *P. sylvestris* canopy reached a height of 14.3 m with a density of about 800 trees ha^−1^ and a leaf area index (LAI) of 1.9 m^2^ m^−2^ in 2003 (Schindler *et al*. 2006). No silvicultural management treatment, such as thinning, has occurred since then. Between 2003 and 2014 the *P. sylvestris* canopy grew to a height of 17–18 m and levelled to 19 m height from 2014 to 2023. On site, a dense understorey of broadleaved species has developed, primarily since 2015 with an average height of 10 m in 2023, leading to an LAI under the dense understorey of up to 5.05 m^2^ m^−2^ in June 2023. The LAI of the whole forest in 2021 (2.81 m^2^ m^−2^), 2022 (2.66 m^2^ m^−2^) and 2024 (2.60 m^2^ m^−2^) (Plapp *et al*. 2025) increased compared to 2003. Yet, the forest is still composed of two layers: larger *P. sylvestris* trees and several different deciduous tree species in the second layer (from here on ‘understorey’) (Fig. 1). The main species in the understorey are *Tilia* spp. (*Tilia cordata* Mill. and *Tilia platyphyllos* Scop.) (32% of understorey trees), *Carpinus betulus* L. (24%) and *Fagus sylvatica* L. (15%) (Plapp *et al*. 2024b), which are all species capable of high photosynthesis rates under full sunlight conditions (e.g. Legner *et al*. 2013). As a consequence of the 2018 hot drought, >60% of *P. sylvestris* trees had died until 2023, which is an increase of ~13% compared to tree mortality in September 2020 (Haberstroh *et al*. 2022). In 2023, the stand density of *P. sylvestris* declined to 375 trees ha^−1^ of which 116 trees ha^−1^ were considered standing dead trees (Plapp *et al*. 2024b). Dead trees were not felled until they had fallen or endangered infrastructure. Until winter 2023/2024, dead wood remained on the ground (Fig. 1).

Due to its location in the floodplain of the Rhine valley, the sediment consists of a carbonate‐rich (pH = 7.8), two‐layer Haplic Regosol (Goffin *et al*. 2014). As a result of historical river regulation of the nearby Rhine between 1817 and 1959, the groundwater level is around 7 m (Brandes *et al*. 2007) and not within reach of tree roots.

### Meteorological measurements

In‐canopy air temperature (*T*_*air*_) at DE‐Har was measured 2 m above ground level at the base of the tall tower with an actively ventilated psychrometer using a PT1000 based on the design of Frankenberger (1951). Precipitation was recorded on top of the small tower at a height of 18 m above the ground using a heated ombrometer Model 5.4031 (Adolf Thies, Göttingen, Germany). Although the *P. sylvestris* canopy is at 17–19 m, no trees with such a canopy height grew in close vicinity to the precipitation measurement on the tower, which was unaffected by trees (Fig. S1). Vapour pressure deficit (VPD) was measured at 27 m on the tall tower above the canopy using a CS215 temperature/humidity probe in a passively ventilated screen (Campbell Scientific, Logan, UT, USA). Shortwave irradiance was measured using a thermopile pyranometer (CM21; Kipp & Zonen, Delft, Netherlands) at 30 m on the tall tower. Volumetric water content reported in this experiment was taken as the average of six sensors inserted in two spatially separated profiles in 0.05, 0.10 und 0.20 m depth using CS655 soil sensors (Campbell Scientific) according to ICOS instructions (Op de Beeck *et al*. 2017).

### Ecophysiological measurements 2023

To compare the current ecophysiological response of *P. sylvestris* with deciduous understorey trees in 2023, *C. betulus*, as one of the dominant understorey tree species (24% of all broadleaf understorey trees; Plapp *et al*. (2024b)), was chosen due to its proximity to the canopy access tower and accessibility of the tree crown for water potential measurements, similar to the studied *P. sylvestris* trees. Both species had been monitored already during the 2018 hot drought for water potentials (Haberstroh *et al*. 2022). Water potential of *P. sylvestris* (n = 5) and *C. betulus* (n = 6) was measured pre‐dawn (*Ψ*_*PD*_) (before sunrise, 04:00–06:00 CEST) on terminal branchlets, representing leaf water potentials, using a Scholander‐type pressure chamber (1505D; PMS Instrument, USA) (Scholander *et al*. 1965). Branchlets were cut with long‐reach pruning shears from the ground or from canopy access towers. Measurements were taken on six different days between April and September 2023.

Sap flow sensors (SFM1; ICT International, Armidale, Australia) were installed on *P. sylvestris* (n = 7) and *C. betulus* (n = 6) in early May 2023. Sensors were placed in the trunk 1.20 m above ground and consisted of three 35 mm stainless‐steel needles. Measurements were taken at two sapwood depths: at 12.5 (outer xylem) and 27.5 mm (inner xylem) below the cambium. All trees had a sapwood depth > 27.5 mm. Heat pulses (30 J) were generated by a heating element in the middle needle every 20 min; data were stored on internal loggers. Calculation of sap flux density (*J*_*s*_) followed the Heat Ratio Method (Burgess *et al*. 2001). Total *J*_*s*_ was determined by averaging *J*_*s*_ in the two different measurement depths for both species. To account for nocturnal sap flow, *J*_*s*_ was corrected for offsets from the baseline on nights with unlikely environmental conditions for nocturnal sap flow (photosynthetically active photon flux density <5 μmol m^−2^ s^−1^, VPD <0.05 kPa, *T*_*air*_ >0°C, RH > 95%) between May and November 2023. Due to sensor failure, *J*_*s*_ of three *P. sylvestris* trees was removed from the dataset starting from 24/08/23, 06/09/23 and 21/09/23. For comparison, max. *J*_*s*_ of *P. sylvestris* measured at DE‐Har were derived from Brandes *et al*. (2007) for the years 2003 and 2004.

Selected *P. sylvestris* trees had a height of 16.6 ± 0.4 m and a diameter at breast height (DBH) of 24.8 ± 1.1 cm. *C. betulus* trees were smaller (10.7 ± 0.3 m) and had a lower DBH (16.8 ± 0.4 cm). Note that not all *P. sylvestris* trees measured for sap flow were accessible for water potential measurements.

### Enhanced vegetation index (EVI) and phenocams

Vegetation indices, such as the enhanced vegetation index (EVI) are an ideal tool to track ongoing changes in vegetation abundance and seasonality (Huete *et al*. 2002). Values for EVI typically vary between 0 and 1, where higher EVI values denote greener vegetation than lower EVI values (Swain *et al*. 2017). EVI is responsive to variations in canopy structure, such as LAI, canopy type or architecture (Gao *et al*. 2000; Huete *et al*. 2002), which naturally vary between deciduous‐ and conifer‐dominated forests. For other vegetation indices, such as the normalized difference vegetation index (NDVI), it has been shown that deciduous‐dominated forests have higher maximum values than coniferous forests in summer (e.g. Soudani *et al*. 2012) and show clearer differences in reflectance from summer to winter than evergreen species due to a leafless period (Wang *et al*. 2023). Data for EVI was downloaded and analysed from the Moderate‐resolution Imaging Spectroradiometer (MODIS; MOD13Q1 V6.1; Didan 2021) via the Google Earth Engine at a spatial resolution of 250 m. For the exact calculation of EVI, please see Methods S1, equations S1 and S2 in the Supporting Information—S1. Data for DE‐Har were centred on the tall tower location and cover an area of 62,500 m^2^ (250 × 250 m). Thus, the area covered by MODIS is larger than the actual field site. However, as the actual research plot is embedded within a larger *P. sylvestris* forest, it can be assumed that EVI data with a resolution of 250 m are sufficient to detect changes at the field site. Likewise, the EVI resolution fits the eddy covariance footprint well: during daytime (shortwave radiation >10 W m^−2^) 70% of the fluxes originate from an area, which covers ~54,000 m^2^ and 80% from an area which covers ~125,000 m^2^.

One data point was available for every 16‐day period and averaged per month from 2000 to 2023. If all input pixels were cloudy, a computation based on a historical database occurred (Didan 2021). Nevertheless, we filtered data, which were lower than the 5% confidence interval in winter to account for unrealistically low values caused by extreme winter conditions. Differences between evergreen and deciduous vegetation are especially pronounced in winter and summer. Thus, we calculated average values for EVI between July and August (summer), as well as for December to February (winter). In winter, EVI can be influenced by snow cover, however differences between vegetation types should still be visible (cf. Wang *et al*. 2023). In addition, we calculated seasonal courses of EVI for pre‐ (2000–2017) and post‐2018 years (2019–2023) to depict monthly changes in EVI after 2018.

Two phenocams (Star Dot NetCam SC 5MP D/N; Star Dot Technologies., Buena Park, CA, USA) have been continuously operated since 2018 at the tall tower, taking recordings of the canopy (29.6 m) and understorey (8.4 m) facing towards North at 30‐min intervals. Pictures can be accessed from the phenocam network (see data availability statement). From the cameras, green chromatic coordinate values (G_CC_) are calculated for selected regions of interests according to Richardson *et al*. (2018), which have been shown to closely track leaf phenology (Sonnentag *et al.*, 2012). We chose the 90th percentile across a 3‐day moving window to minimize the effects of atmospheric variations on G_CC_ values (Richardson *et al*. 2018; Seyednasrollah *et al*. 2019). Finally, we extracted the dates of the maximum yearly value of G_CC_ (90th percentile) and the date where 10% and 50% of this maximum value was reached (Richardson *et al*. 2018; Seyednasrollah *et al*. 2019), to depict the approximate timing of leaf flush of the understorey, that is, the time point when the deciduous understorey became photosynthetically active (Dougherty *et al*. 1979; Greco & Baldocchi 1996).

### Ecosystem carbon fluxes

Ecosystem CO_2_ fluxes between the forest and the atmosphere are available from eddy covariance from 2003 to 2006 and from 2019 to 2023. Details on the instrumentation and data processing of the eddy covariance data from 2005 are described in Holst *et al*. (2008). The measurement set‐up and data processing from 2019 to 2023 are identical to Haberstroh *et al*. (2022). The set‐up consists of a CSAT‐3 ultrasonic anemometer‐thermometer (Campbell Scientific) co‐located with a closed‐path infrared gas analyser (Li‐7200; Licor, Lincoln, NB, USA) on the tall tower at a height of 27 m (Fig. S1). Fluxes were processed in 30‐min blocks using Eddy‐Pro. Data with quality control flags 0 and 1 and a flow rate >12 l min^−1^ are further used in this study. Data availability of measured CO_2_ fluxes was 59% (2019), 64% (2020), 73% (2021), 71% (2022) and 77% (2023). CO_2_ fluxes under low turbulence were removed using an iterative friction velocity threshold (Papale *et al*. 2006). Missing CO_2_ fluxes were then gap‐filled following Reichstein *et al*. (2005) using REddyProc (v. 1.3.3; Wutzler *et al*. 2018). With REddyProc, fluxes were partitioned into gross primary productivity (GPP; Fig. S2A) and ecosystem respiration (R_eco_; Fig. S2B) based on nighttime fluxes (Reichstein *et al*. 2005) and data from air temperature at 2 m and above‐canopy short‐wave irradiance.

It should be noted that Holst *et al*. (2008) used an open‐path sensor in their study compared to the closed‐path sensor used in the data from 2019 to 2023. Open‐path sensors can be subject to carbon flux underestimation in winter in cold climates, due to artificial heating of the instruments surface (Burba *et al*. 2008). While in Holst *et al*. (2008) there was no correction applied for instrument‐related sensible heat flux (Burba *et al*. 2008), we are confident that potential errors in NEE are small. Carbon flux winter underestimation is a major problem when *T*_*air*_ drop <−10°C (Cassidy *et al*. 2016), which was rarely the case at DE‐Har. In 2005, only on 6 days min. *T*_*air*_ reached values <−10°C and only 6 days reached average *T*_*air*_ < −5°C (Christen *et al*. 2024b). Similarly, differences between open‐path and closed path sensors are only a major issue at *T*_*air*_ < −10°C and negligible at *T*_*air*_ between 0 and 20°C (Cassidy *et al*. 2016, supplement).

### Assessment of legacy effects on net ecosystem carbon exchange

The analysis of legacy effects requires a predefined baseline without marked stress conditions against which data can be assessed (Müller & Bahn 2022). Unfortunately, no data for ecosystem carbon fluxes were available from 2007 to 2018 (Table 2), due to lack of resources, staff and instrumentation. However, remote sensing products, such as the NDVI and EVI, which are often used as proxy for gross primary productivity (GPP) (e.g. Sims *et al*. 2006), provide evidence that the *P. sylvestris* forest was in a rather stable condition before 2018 (Haberstroh *et al*. 2022). Thus, we decided to compare NEE of post 2018 years against NEE of 2005, which depicted optimal growth conditions (Holst *et al*. 2008) with growing season (April–September) *T*_*air*_ (15.8°C, −0.3 K relative to 1991 climatic reference period) and precipitation (381 mm, −10 mm) resembling growing season averages (1991–2020), thus stressing its suitability as baseline to assess legacy effects. Other available years, such as 2003/2004 or 2006 were characterized by distinct summer drought periods (Schindler *et al*. 2006; Holst *et al*. 2008). As Holst *et al*. (2008) did not partition NEE in GPP and R_eco_, we used NEE for this comparison. We calculated monthly deviations in NEE from 2005 as in equation 1:

(1) $$\Delta_{\mathrm{NEE}2005}={\mathrm{NEE}}_{X}-{\mathrm{NEE}}_{2005}$$

where NEE_X_ is monthly NEE of the years 2019, 2020, 2021, 2022 and 2023, and NEE_2005_ is the corresponding monthly NEE measured in the year 2005.

Analogous to equation 1, we calculated the difference in NEE of the year 2021 to NEE of all years post‐2018 (Δ_NEE2021_). 2021 was the only year after 2018 with above average precipitation (693 mm, +47 mm) and below average *T*_*air*_ (9.9°C, −0.7 K). Thus, it is the only year post‐2018, which qualifies as baseline, according to Müller & Bahn (2022).

### Statistical analysis

To compare the ecophysiological behaviour of both species in 2023, linear mixed effect models (*lmer*) (*lme4*, Bates *et al*. 2015) were calculated for the response variables *Ψ*_*PD*_ and *J*_*s*_. For *Ψ*_*PD*_, species and day of year, and for *J*_*s*_ species were treated as fixed effect; TreeID was set as random effect in both models. For *J*_*s*_, a second model was conducted to compare *J*_*s*_ at the two different xylem depths per species. Significant differences in monthly EVI before and after 2018 were detected using a linear mixed effect model (*lmer*) with the response variable ‘EVI’ predicted by the variable ‘month’ and ‘period’ (pre‐ and post‐2018). Year was set as random effect. Significant differences in all of these models were assessed with pairwise comparisons (Tukey adjustment) with the function *emmeans* (Lenth 2024) in R (R Core Team 2022). *Ψ*_*PD*_ was square‐root transformed to meet model assumptions. For all *J*_*s*_ models, model assumptions were not met, but model results were confirmed with a non‐parametric Wilcox test.

Monthly Δ_NEE2005_ was correlated with average monthly *T*_*air*_ and maximum monthly VPD (VPD_max_) as proxies for atmospheric drought, and with average monthly VWC and monthly precipitation sum as proxies for edaphic drought. The relationship of Δ_NEE2005_ and environmental parameters was assessed with linear regressions (*lm*) in R (R Core Team 2022). For these regressions, only data points where monthly mean *T*_*air*_ exceeded 15°C were used to isolate months with a high likelihood of atmospheric and/or edaphic drought conditions. For 2019, no reliable VWC data were available on a monthly basis due to instrument communication failures. Likewise, 2 months in 2021 (June and July) were removed from the analysis due to similar technical problems with VWC. Assumptions for all models were checked with the Shapiro–Wilk test (normal distribution of residuals) and the function *simulateResiduals* in the package *DHARMa* (Hartig 2022).

The relationship of Δ_NEE2021_ and environmental parameters (assessed with piecewise two segment linear regressions with the function *segmented* for *T*_*air*_, VPD_max_ and VWC (*segmented*, Fasola *et al*. 2018) and linear regression for precipitation) can be found in the Figs. S4 and S5.

## RESULTS

### Meteorological conditions between 2018 and 2023

**Fig. 2:**
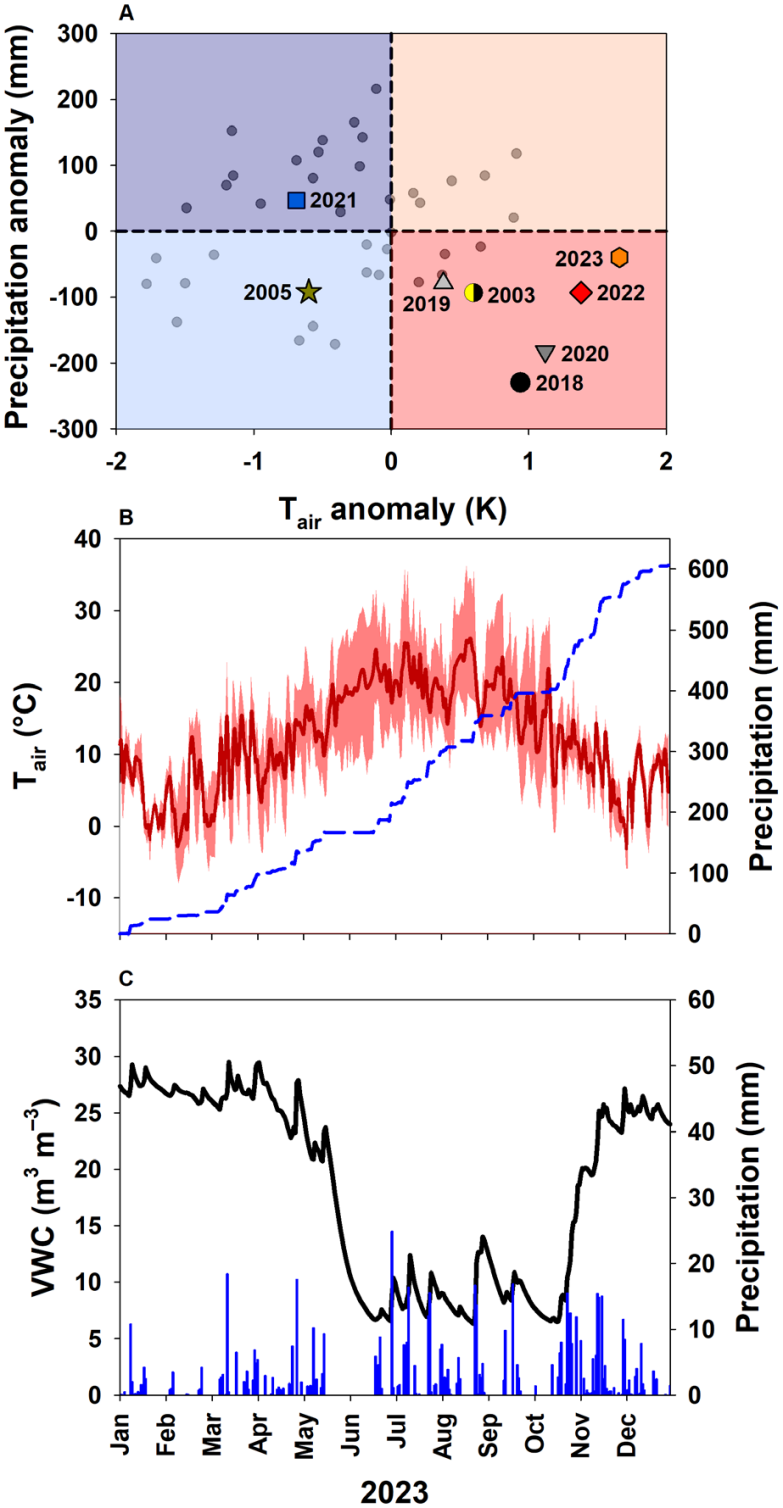
Yearly in‐canopy air temperature (*T*_*air*_) and above‐canopy precipitation anomaly to the long‐term climate reference period (1991–2020) of the years 2003, 2005 and 2018–2023 (A). All other years since 1978 are illustrated by points in the background. Mean air temperature (*T*_*air*_) with minimum and maximum *T*_*air*_, cumulative precipitation (B), volumetric soil water content (VWC) in the top 0.20 m and precipitation (C) for the year 2023.

2018 was the driest year at DE‐Har, with only 417 mm of precipitation (−230 mm, −36%) compared to the long‐term annual precipitation of 646 mm in the 1991–2020 reference period (Fig. 2A). All following years, except 2021, were drier and warmer than the 1991–2020 reference period, with conditions in 2020 (463 mm, −183 mm, −28%) being almost as dry as 2018. 2022 was characterized by both, above‐average in‐canopy *T*_*air*_ (12.0°C, +1.4 K) and below average precipitation (553 mm, −93 mm, −14%). Only 2021 was colder (9.9°C, −0.7 K; 693 mm, +47 mm, +7%) than the long‐term average (1991–2020). In contrast, 2023 was the hottest year (+12.3°C, +1.7 K) on record for the investigated ecosystem, with only minor deviations from the long‐term precipitation mean (−40 mm, −6%, 1991–2020). In particular, 2023 was characterized by a hot summer with two distinct periods of high *T*_*air*_ in July (*T*_*max*_: 36.0°C) and August 2023 (*T*_*max*_: 36.2°C) (Fig. 2B). Topsoil moisture (first 0.20 m) declined in May and early June, but did only recover to pre‐drought levels in mid‐November despite frequent precipitation during summer (Fig. 2C).

### Ecophysiological tree responses in 2023

**Fig. 3:**
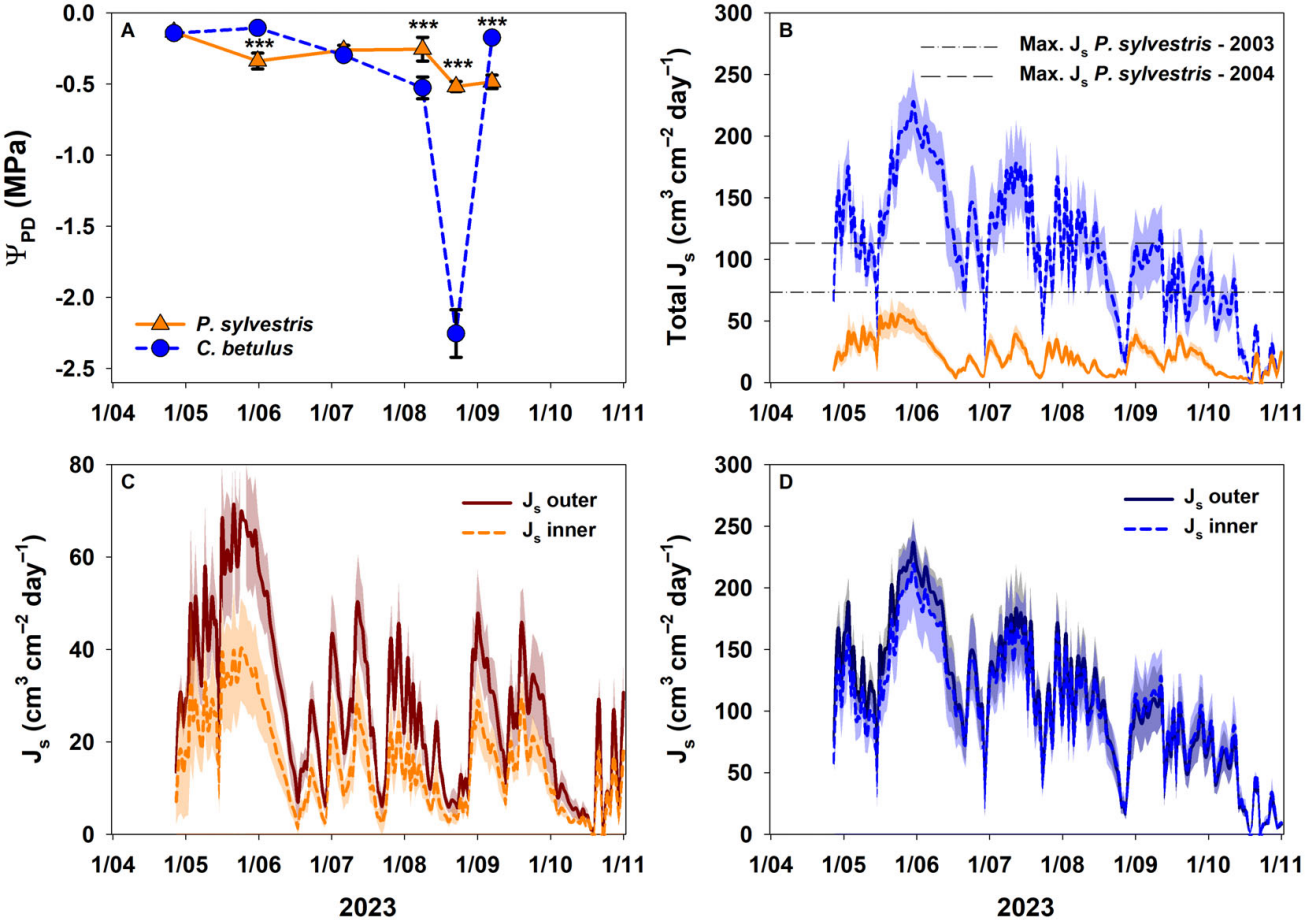
Pre‐dawn water potential (*Ψ_PD_*) ± 1 SE for *Pinus sylvestris* (n = 5) and *Carpinus betulus* (n = 6) between May and September 2023 (A). Total daily sum of sap flux density (*J*_*s*_) ± 1 SE for *P. sylvestris* (n = 7) and *C. betulus* (n = 6) (B) with max. *J*_*s*_ for *P. sylvestris* in 2003 and 2004 (Brandes *et al*. 2007) and daily sum of sap flux density (*J*_*s*_) ± 1 SE in the inner and outer xylem for *P. sylvestris* (n = 7) (C) and *C. betulus* (n = 6) (D) between May and November 2023. Significance differences between species in panel (A) is given at *P* < 0.001***. Please note the different scale of panel (C) to panels (B) and (D).

Pre‐dawn water potentials (*Ψ*_*PD*_) were similar between both investigated species in May and July 2023, but significantly differed in June (*p* < 0.001) with higher values for *C. betulus* (Fig. 3A). From early August onwards, only a slight drop in *Ψ*_*PD*_ from −0.26 ± 0.08 MPa to −0.52 ± 0.03 MPa occurred for *P. sylvestris*, while *Ψ*_*PD*_ of *C. betulus* differed significantly (*p* < 0.001), decreasing strongly from −0.53 ± 0.08 MPa to −2.26 ± 0.17 MPa. In September, *Ψ*_*PD*_ of.

*C. betulus* recovered to values close to 0 MPa, while *Ψ*_*PD*_ of *P. sylvestris* remained at values of −0.49 ± 0.05 MPa (*p* < 0.001).

Sap flux density (*J*_*s*_) of both species demonstrated vast and persistent differences between species water fluxes in 2023 (*p* < 0.001). *J*_*s*_ of *P. sylvestris* rarely exceeded 50 cm^3^ cm^−2^ day^−1^ in spring (max.: 55.6 ± 13.7 cm^3^ cm^−2^ day^−1^), which is lower than *J*_*s*_ measured in 2003 and 2004 (Fig. 3B, Brandes *et al*. 2007). *J*_*s*_ of *P. sylvestris* nearly ceased in the first dry‐down phase in June (Fig. 3B) and remained at a low level until the end of the year. Over the whole measurement period, *J*_*s*_ in the inner xylem was significantly reduced by 49% on average compared to *J*_*s*_ in the outer xylem (*p* < 0.001) (Fig. 3C). *J*_*s*_ of *C. betulus* peaked in spring (228.1 ± 26.7 cm^3^ cm^−2^ day^−1^), and was four times higher than *J*_*s*_ of *P. sylvestris* throughout the summer. After the first dry‐down in June, *J*_*s*_ recovered to values exceeding 150 cm^3^ cm^−2^ day^−1^ until the end of August, when *J*_*s*_, as well as *Ψ*_*PD*_ of *C. betulus* dropped strongly, but recovered quickly in September (Fig. 3B). For *C. betulus*, *J*_*s*_ of the inner and outer xylem were also significantly different (*p* < 0.05). Until September, outer *J*_*s*_ exceeded inner *J*_*s*_, while this pattern was reversed afterwards (Fig. 3D).

### Enhanced vegetation index (EVI) and green chromatic coordinate (G_CC_ ) values

**Fig. 4:**
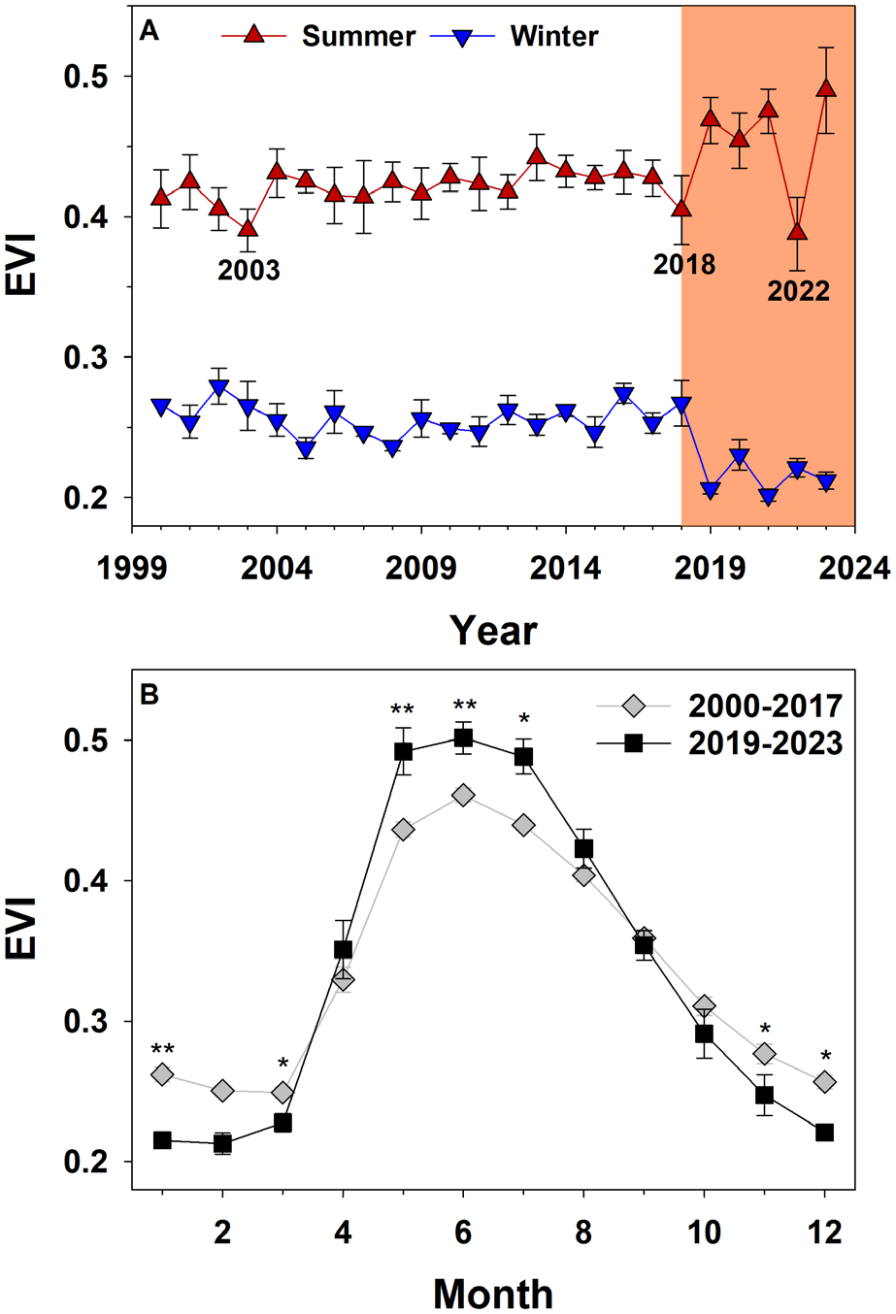
Enhanced vegetation index (EVI) of the *P. sylvestris* ecosystem at a spatial resolution of 250 m centred on the main tower location at DE‐Har from 2000 to 2023 for summer (July and August) and winter (December–February) ± 1 SE (A) and seasonal course of EVI for the periods 2000–2017 and 2019–2023 (B). Significant differences between months given at *P < 0.05, **P < 0.01.

Long‐term records of EVI show that from 2000 to 2017, the summer EVI of the ecosystem at DE‐Har was relatively stable with values ranging between 0.405 ± 0.015 and 0.442 ± 0.016 (Fig. 4A). Only in 2003, an extreme dry year, a drop in EVI (0.390 ± 0.015) was evident. Similarly, during the 2018 drought, values decreased to 0.404 ± 0.024 (Fig. 4A). Interestingly, after 2018, an increase in summer EVI to 0.490 ± 0.031 (2023) was observed, with the exception of the extreme drought in 2022 (0.388 ± 0.026). Contrarily, in winter, EVI strongly dropped from 0.267 ± 0.016 after 2018 to values of 0.219 ± 0.004 in 2023 (Fig. 4A). Seasonal courses of EVI before and after 2018 confirm these seasonal dynamics (Fig. 4B). In January, March, November and December, EVI significantly decreased after 2018 (*p* < 0.05), while a significant increase could be observed from May to July (*p* < 0.05).

From green chromatic coordinate (G_CC_) values, it is evident that leaves of deciduous understorey trees started to develop in late March, reaching 50% of the max. G_CC_ value in mid‐April (Table 1). In early May, G_CC_ values reached their annual max. values, indicating full leaf development. Variations within years were small, however leaf development in 2019, after the 2018 hot drought, and in 2021, a particularly cold and wet year, were slower than in 2020, 2022 and 2023 (Table 1).

**Table 1:** Dates for green chromatic coordinate (G_CC_) values (90th percentile), when 10% and 50% of the maximum G_CC_ was reached (transition date) and when the maximum G_CC_ was reached per year. These dates indicate the timing of the leaf flush and development of understorey trees at DE‐Har (phenocam at 8.4 m – hartheim2).

Year	2019	2020	2021	2022	2023
Transition date (10%)	28/03	26/03	30/03	29/03	25/03
Transition date (50%)	15/04	9/04	21/04	16/04	14/04
Maximum G_CC_	20/05	1/05	11/05	5/05	5/05

### Ecosystem carbon fluxes

**Fig. 5:**
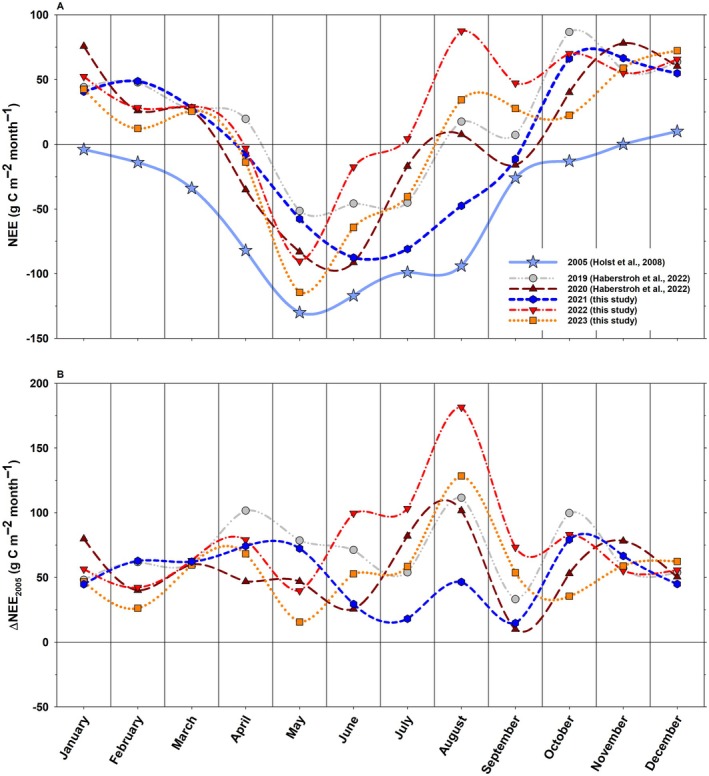
Monthly net ecosystem carbon exchange (NEE) of the *P. sylvestris* ecosystem for the years 2005 and 2019–2023 (A) and monthly difference in NEE of 2005 (Δ_NEE2005_) to the years 2019–2023 (B).

Before the 2018 drought, the ecosystem was a considerable carbon sink with average values for NEE of −391 ± 204 g C m^−2^ year^−1^, including dry years, such as 2003 or 2006 (Schindler *et al*. 2006; Holst *et al*. 2008) (Table 2). In 2005, the predefined baseline, NEE reached values of −603 g C m^−2^ year^−1^. Carbon uptake was especially high during the vegetation period, however, also in winter the ecosystem was close to carbon neutral (Fig. 5A). In the past, during single dry and hot years, such as 2003 (Fig. 2A), and 2006, which was characterized by high summer air temperature, the ecosystem was still a significant carbon sink (Table 2).

**Table 2:** Net ecosystem carbon exchange (NEE), gross primary productivity (GPP) and ecosystem respiration (R_eco_) for the *Pinus sylvestris* forest at DE‐Har for 2003–2006 and 2019–2023 with difference of NEE to NEE of the year 2005 (ΔNEE_2005_).

Year	NEE (g C m^−2^ year^−1^)	ΔNEE_2005_ (g C m^−2^ year^−1^)	GPP (g C m^−2^ year^−1^)	R_eco_ (g C m^−2^ year^−1^)	references
2003/04	−195	–	–	–	Schindler *et al*. (2006)
2005	−603	–	–	–	Holst *et al*. (2008)
2006	−376	–	–	–	Holst *et al*. (2008)
2019	229 ± 18	+832	1,403 ± 141	1,632 ± 128	This study
2020	71 ± 15	+674	1,418 ± 24	1,489 ± 14	This study
2021	13 ± 28	+616	1,561 ± 107	1,573 ± 130	This study
2022	329 ± 19	+932	1,287 ± 75	1,616 ± 89	This study
2023	63 ± 1	+666	1,909 ± 199	1,973 ± 199	This study

By comparing NEE of 2005 and 2021 (+13 ± 28 g C m^−2^ year^−1^), both years without marked stress conditions during the growing season (Figs. 2A, 5A), it becomes evident that the ecosystem has lost a considerable proportion of its carbon sink capacity (Δ_NEE_ = +616 g C m^−2^ year^−1^; Table 2). Compared to 2005, NEE decreased in all months post 2018 (positive Δ_NEE2005_) (Fig. 5B). Between November and February, this difference was quite stable at (~ + 50 g C m^−2^ month^−1^), but increased strongly thereafter, peaking in August (except 2021) with the largest difference observed in August 2022 (Δ_NEE2005_ = +181 g C m^−2^ month^−1^) (Fig. 5B). This large difference in August 2022 was mostly driven by a large drop in GPP (Fig. S2). The largest yearly difference to 2005 was also reached in the hot and dry year 2022, with values of +932 g C m^−2^ year^−1^ (Δ_NEE2005_) (Table 2). Notably, GPP has continuously increased since 2019, with the exception of 2022 (Table 2, Fig. S2), reaching a maximum of 1,909 ± 199 g C m^−2^ year^−1^ in 2023. These patterns fit the overall increase in EVI (Fig. 4), which is depicted by the highly significant correlation between GPP and EVI (*p* < 0.001, *R* ^2^ = 0.91) (Fig. S3). However, R_eco_ similarly increased with EVI (*p* < 0.001, *R* ^2^ = 0.70) (Fig. S3), partially offsetting the increase in GPP (Fig. S2B, Table 2). In 2023, R_eco_ reached a maximum of 1,973 ± 199 g C m^−2^ year^−1^.

These patterns in GPP and R_eco_ led to the ecosystem being a (strong) carbon source, for example, in 2022 (+329 ± 19 g C m^−2^ year^−1^), but also in the comparably wet, but hot, 2023 (+63 ± 1 g C m^−2^ year^−1^), with 2020 (+71 ± 15 g C m^−2^ year^−1^) and 2019 being in between (+229 ± 18 g C m^−2^ year^−1^) (Fig. 5A and Table 2).

**Fig. 6:**
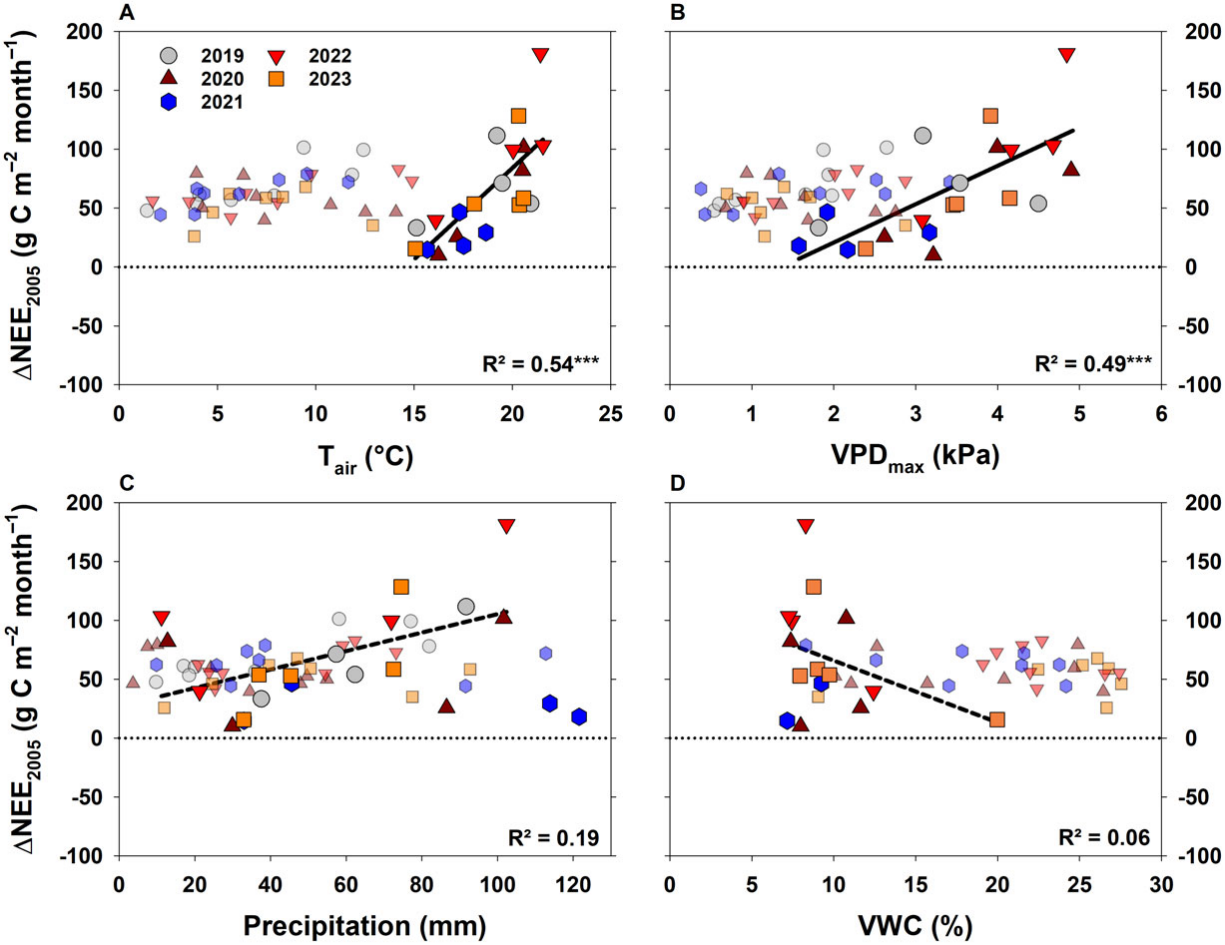
Relationship of monthly difference in net ecosystem exchange (NEE) of 2005 (Δ_NEE2005_) to the years 2019–2023 and monthly mean air temperature (*T*_*air*_) (A), monthly maximum vapour pressure deficit (VPD_max_) (B), monthly precipitation sum (C) and monthly mean soil volumetric water content (VWC) (D). The same analysis for Δ_NEE2021_ can be found in Fig. S4 in the Supporting Information—S1. For linear regressions in panels A, B, D, only data from months with a mean *T*_*air*_ > 15°C were used. Significance at *P* < 0.001***. Large symbols in panels A, B and D were used for the linear regressions.

As the ecosystem was exposed to recurrent drought and heatwaves after 2018, all post‐drought years (except 2021) were drier and hotter (2019, 2020, 2022, 2023) than the long‐term average (Fig. 2A). Fig. 6 illustrates the impact of these conditions on Δ_NEE2005_. Especially under atmospheric (and edaphic) drought conditions, NEE significantly decreased in comparison to 2005, that is, with rising *T*_*air*_ and VPD_max_ (*p* < 0.001), but also with declining VWC (although not significant) (Fig. 6A, B, D). However, this decrease in NEE was only evoked after a certain threshold was exceeded (*T*_*air*_ > 15°C). Under colder and wetter conditions, Δ_NEE2005_ did not show a clear pattern with *T*_*air*_, VPD_max_ and VWC and fluctuated at values of Δ_NEE2005_ ~ +50 g C m^−2^ month^−1^ with some minor exceptions. In addition, NEE declined non‐significantly with increasing precipitation sum (Fig. 6C). However, some of these large precipitation events co‐occurred as convective precipitation events with high *T*_*air*_ in the summer months, such as in August 2019, 2020 or 2022. All of the above‐mentioned patterns with environmental parameters, but especially VWC, were confirmed and strengthened when 2021 instead of 2005 was used as baseline. Here, NEE strongly decreased in comparison to 2021 when the following thresholds were crossed: *T*_*air*_ > 16.1 ± 1.0°C, VPD_max_ >2.4 ± 0.4 kPa, VWC < 10.1 ± 1.0% (Fig. S4, segmented linear regressions).

**Fig. 7:**
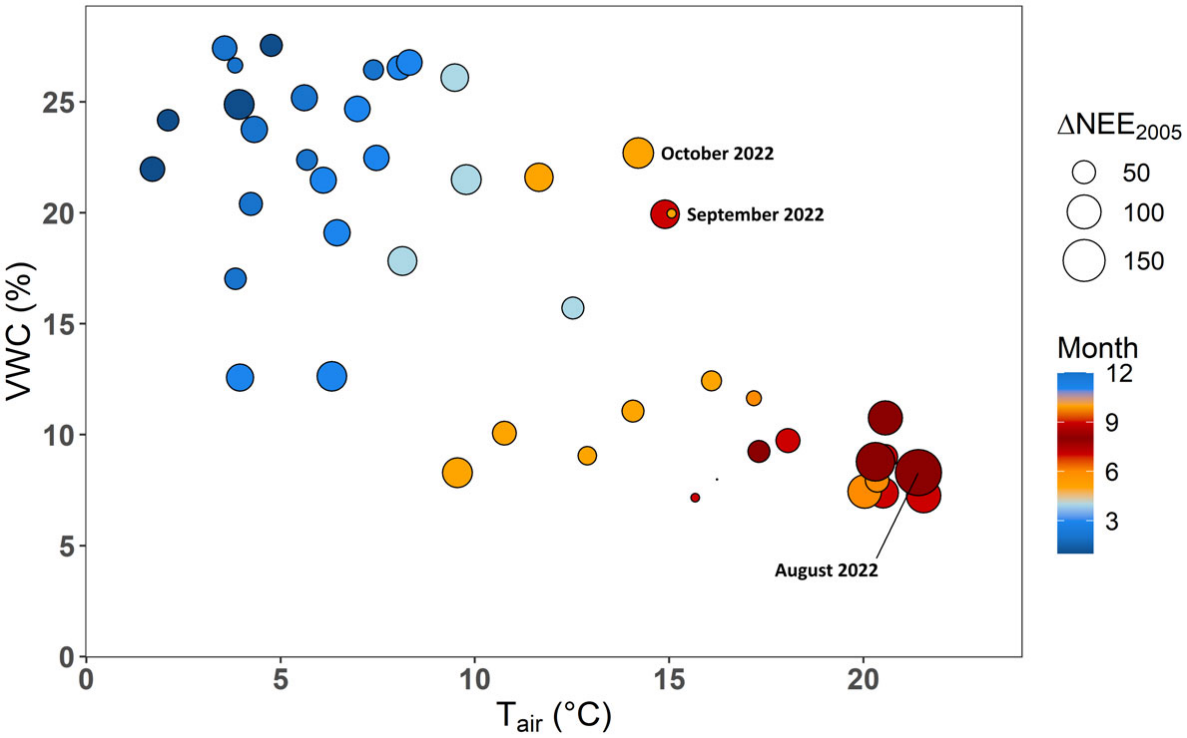
Monthly difference in net ecosystem exchange (NEE) of 2005 (Δ_NEE2005_) to the years 2020–2023, monthly mean air temperature (*T*_*air*_) and monthly mean volumetric soil water content (VWC). Larger circles indicate more positive Δ_NEE2005_. Months are colour‐coded from 1 (January) to 12 (December). Different months are illustrated with a colour scale. The same analysis for Δ_NEE2021_ can be found in Fig. S5 in the Supporting Information—S1.

In conclusion, the decrease in monthly NEE compared to 2005 (and 2021) was stronger with both atmospheric and edaphic droughts, which complicates assessment of the individual impact of both stressors on Δ_NEE2005_. Yet, Fig. 7 and Fig. S5 depict that NEE especially decreased in comparison to 2005 (or 2021) when both stressors co‐occurred in a compound event, that is, high *T*_*air*_ and low VWC. These compound events preferentially materialized in the summer months and led to a strong carbon release from the *P. sylvestris* forest. In Fig. 7, 2 months at moderately high *T*_*air*_ (14.2°C and 14.9°C), but high VWC (19.9% and 22.7%) stand out. These months (September and October 2022) followed the extreme drought and heat month of August 2022, where the difference in NEE from 2005 was highest in the study period (Δ_NEE2005_ = +181 g C m^−2^ month^−1^) (Figs. 5B and 7). The winter months (blue points) did not significantly contribute to deviations in NEE from 2005 (Figs. 5 and 7). Similar patterns were found when Δ_NEE2021_ instead of Δ_NEE2005_ was used (Fig. S5).

## DISCUSSION

The occurrence of recurrent hot‐dry events (*compound droughts*) and concurrent drought legacy effects severely threaten ecosystem processes, functioning and survival. In our *P*. *sylvestris* forest in SW Germany (DE‐Har), ecophysiological parameters, EVI and NEE, indicate that this particular ecosystem is suffering severely on all assessed scales. We attribute this to the legacy effects of the 2018 hot drought, combined with the dry and hot conditions that preceded and followed it. The forest has lost a considerable capacity for carbon uptake due to high *P. sylvestris* mortality, which induced a vegetation shift towards a more deciduous‐dominated forest. Although GPP did steadily increase after 2019, the ecosystem has mainly become a net source of carbon dioxide due to a subsequent increase of R_eco_, exacerbated by recurrent atmospheric and edaphic droughts.

### Progressive shift in species dominance as legacy effect of (recurrent) hot droughts with persistent impact on net ecosystem carbon exchange

Data from ecophysiological measurements in 2023 indicate that *P. sylvestris* trees were rather inactive with low tree water fluxes. While this is in line with its isohydric behaviour (Poyatos *et al*. 2008) and the dry growing season in 2023 (−81 mm of precipitation compared to 391 mm between 1991 and 2020), *J*_*s*_ of *P. sylvestris* at DE‐Har was higher in the extreme drought year 2003 (Brandes *et al*. 2007) (−129 mm of precipitation compared to 319 mm between 1991 and 2020 in the vegetation period). As needle water potentials of *P. sylvestris* dropped to −7.5 MPa in 2018 (Haberstroh *et al*. 2022) and *Ψ*_88_ values (−5.70 MPa; Martínez‐Vilalta *et al*. 2009) were crossed, it is likely that xylem conductivity decreased, for example, through embolism formation (Lüttschwager & Remus 2007; Haberstroh *et al*. 2022). The reduction of ~50% in *J*_*s*_ in the inner xylem might be species‐specific for *P. sylvestris* (Köstner *et al*. 1996; Cermak & Nadezhdina 1998; Nadezhdina *et al*. 2002). In contrast, the deciduous *C. betulus*, exhibited water‐spending behaviour with high *J*_*s*_ in the inner and outer xylem even during dry and hot periods in 2023. Signs of stress in *C. betulus* were only found in late August, but trees recovered quickly in September (cf. Haberstroh *et al*. 2025). This indicates the understorey vegetation is more vital and resilient than the former dominant *P. sylvestris* (Haberstroh *et al*. 2022). Most probably, understorey trees still profited from microclimatic buffering (Zellweger *et al*. 2020; Werner *et al*. 2021), such as lower solar irradiance, maximum *T*_*air*_ and increased cooling effects of evapotranspiration (Zellweger *et al*. 2019; Greiser *et al*. 2024). Concurrently, understorey trees potentially experienced a release from competition (Haberstroh & Werner 2022) of *P. sylvestris* due to ongoing mortality, and a higher resource availability, such as light, water or nutrients (Lloret *et al*. 2012; Gessler *et al*. 2017).

These patterns are well reflected in the dynamics of EVI after 2018, which suggest an up‐greening in summer, but reduction in greenness in winter, indicating a change in canopy type and structure (Gao *et al*. 2000; Huete *et al*. 2002) from a coniferous‐ to a deciduous‐dominated forest (cf. Soudani *et al*. 2012). *P. sylvestris* decline and mortality most likely led to more opening of the upper canopy (Fig. 1), which exposed smaller and younger deciduous understorey vegetation with higher EVI values (cf. Fiore *et al*. 2020) and explains the opposing trend from increasing EVI values und decreasing carbon sink after 2018. Indeed, GPP has been steadily increasing with increasing EVI after the 2018 hot drought (Fig. S3).

From these observed patterns at the species and community scales, it is fair to assume that the contribution of *P. sylvestris* to NEE was rather diminished compared to pre‐2018 conditions, leading to a shift to the younger understorey vegetation. A strong change in seasonal NEE from pre‐ to post‐2018 can be seen in the winter and autumn months, where the ecosystem shifted from carbon sink/neutral to an average carbon source (Fig. 5A). Evergreen coniferous tree species are capable of fixing and using carbon on favourable, sufficiently warm days in these seasons due to their extended growing period (Falge *et al*. 2002; Wang *et al*. 2003; Bowling *et al*. 2024; Diers *et al*. 2024). The G_CC_ values from the understorey Phenocam indicate that leaf flush and development (and thus the capacity to fix carbon) of the deciduous understorey are only fully completed in early May (Table 1), which is also depicted by the large increase in GPP from April to May (Fig. S2). Thus, it is likely that the seasonal changes in NEE are mostly driven by a shift from coniferous to deciduous forest. Potentially, also warmer winter air temperatures have contributed to the increased carbon release in winter (Liu *et al*. 2020; Sanders‐DeMott *et al*. 2020) and should be considered in the future as driving force of winter ecosystem respiration (Van der Woude *et al*. 2023). Nevertheless, it should be noted that, eventually, differences in winter NEE between 2005 (Holst *et al*. 2008) and 2019–2023 might be smaller on certain days than depicted in Fig. 5 due to the missing correction for instrument‐related sensible heat flux (Burba *et al*. 2008) in Holst *et al*. (2008).

All of the above‐discussed patterns and processes led to a severely diminished net capacity of the ecosystem to sequester carbon. This is in line with reported legacy effects on NEE in other ecosystems (e.g. Yu *et al*. 2022; Pohl *et al*. 2023), however the persistent reduction in NEE at DE‐Har compared to pre‐2018 levels, ranging from +616 to +932 g C m^−2^ year^−1^ (Table 2), is particularly strong. Forests are an important terrestrial carbon sink (Pan *et al*. 2011, 2024), but if more forests suffer from such strong reductions in carbon uptake, this invaluable ecosystem service will be severely threatened and potential carbon cycle feedbacks to climate change are likely to occur (diminished land sink). This is in line with the last forest inventory in Germany (Bundeswaldinventur), which found that German forests were an average carbon source between 2017 and 2022 (BMEL 2024), enhanced by disturbances, such as bark‐beetle impacts and high mortality rates.

After such disturbances with high tree mortality, NEE can be strongly impacted by decomposing deadwood (Russell *et al*. 2015; Wijas *et al*. 2024) and increased mineralization processes in the soil (Goulden *et al*. 2011), which are caused by large gaps in the canopy and subsequent increased microbial activity and soil respiration (e.g. Dörr & Münnich 1987; Curiel *et al*. 2007). Such processes would explain the observed increase in R_eco_ since 2019 (Table 1 and Fig. S2). Simultaneously, living biomass is reduced (e.g. by tree mortality) and requires time to reach pre‐disturbance levels (Goulden *et al*. 2011), which is confirmed by increasing GPP since 2019 (Table 1 and Fig. S2). From other studies, it is likely that the ecosystem, despite the shift in vegetation dominance, is developing again into a substantial carbon sink, once reaching a later successional state (Goulden *et al*. 2011; Gough *et al*. 2021), which might even further enhance the carbon uptake capacity as the ecosystem is developing into a multi‐species forest with high structural complexity (Gough *et al*. 2019). However, the development of ecosystem carbon fluxes is strongly dependent on the predominant environmental conditions, which have strongly impacted the ecosystem after 2018.

### Compound events of heat and drought evoke strong ecosystem carbon release

Both atmospheric and edaphic droughts, as stand‐alone stressors, can have significant negative impacts on ecosystem processes, such as water flux or carbon assimilation. Both stressors lead to stomatal closure of plants, if certain thresholds are crossed (e.g. Johnson & Ferrell 1983; Irvine *et al*. 1998; Carminati & Javaux, 2020) to reduce transpirational water losses and reduce the risk of hydraulic failure (McDowell *et al*. 2008; Grossiord *et al*. 2020). Not only is photosynthesis reduced, but under persistent conditions of atmospheric and edaphic drought, plant respiration processes are enhanced (Teskey *et al*. 2015; Scafaro *et al*. 2021). Furthermore, the area of photosynthetic active leaf material is reduced due to premature leaf and needle shedding (Brun *et al*. 2020; Nadal‐Sala *et al*. 2021), as occurred at the studied site in 2018 (Haberstroh *et al*. 2022) and most likely 2022 (Fig. 4A). The interaction of edaphic and atmospheric drought accelerates these negative impacts, as increased atmospheric water demand can initially increase transpirational water losses (McDowell *et al*. 2008; Will *et al*. 2013), which in return causes faster depletion of available soil water resources, exacerbating the severity of the edaphic drought (Swann 2018; Zhou *et al*. 2019; Grossiord *et al*. 2020). These hot‐dry events preferentially materialize and peak in late summer, where a negative impact on the C‐cycle has also been found in other ecosystems (e.g. Pohl *et al*. 2023). This is in line with reports from various ecosystems in Europe during 2018 and 2022, which suffered from severe reductions in NEE during hot‐dry events (Lindroth *et al*. 2020; Smith *et al*. 2020; Thompson *et al*. 2020; Van der Woude *et al*. 2023; Scapucci *et al*. 2024). These extreme conditions can even lead to net carbon losses during the growing season (Fig. 5A), which has rarely been observed before, but could become a regular phenomenon during future extreme climate events (Van der Woude *et al*. 2023).

As the DE‐Har ecosystem was severely affected by the hot drought of 2018 (and previous droughts; Haberstroh *et al*. 2022), it is difficult to disentangle long‐term legacy effects from current year effects (Müller & Bahn 2022), as it is challenging to determine whether the ecosystem has recovered from the previous extreme event before the occurrence of the next extreme event. For example, in other forest ecosystems there are reports of legacy effects of the 2018 hot drought on carbon fluxes in 2019, while NEE returned to pre‐2018 levels in 2020 (Pohl *et al*. 2023). At DE‐Har, 2019 was a year with high carbon losses (+229 ± 18 g C m^−2^ year^−1^), although meteorological conditions only deviated slightly from the long‐term average in the growing season. Thus, it is likely that the ecosystem still suffered from legacy effects of the year 2018 (Haberstroh *et al*. 2022). The delayed recovery of NEE in September and October 2022, despite refilled soil water resources (Fig. 7), similarly raises the question of legacy effects of the year 2022 in 2023. Future observations and longer time‐series of NEE will help to elucidate the legacy size and duration on ecosystem carbon fluxes at DE‐Har and other ecosystems. Furthermore, detailed studies of ecophysiological processes at the individual tree level, such as leaf gas exchange, sap flow and water potential dynamics of all major tree species growing at DE‐Har, will provide a better understanding of the mechanisms underlying the observed changes in ecosystem processes, such as the shift in species dominance and its impact on ecosystem carbon fluxes.

## CONCLUSION

The impacts of the 2018 hot drought and the following years have had substantial negative effects at different scales on the *P. sylvestris* ecosystem at DE‐Har, which are most likely legacy effects of several recurrent hot drought events. This ecosystem is slowly shifting to a deciduous‐dominated forest, which is, however, still mostly a carbon source due to the frequent occurrence of compound droughts in recent years. If climatic extremes do occur at the same frequency as 2018–2023, this could markedly retard or even prevent ecosystem recovery, thus endangering more ecosystems in Central Europe that might turn into permanent carbon sources.

## AUTHOR CONTRIBUTIONS

SH, AC and CW conceived the study. SH, AC, MS and FS collected and analyzed the data. SH, CW and AC interpreted the data. SH wrote the manuscript draft with inputs from AC, MS, FS and CW. All authors contributed critically to the drafts and gave final approval for publication.

## Conflict of Interest

The authors declare no conflicts of interest.

## Supporting information


**Fig. S1.** Illustration of the precipitation and eddy covariance measurement positions at the two towers at DE‐Har. Photo: University of Freiburg.
**Fig. S2.** Monthly gross primary productivity (GPP) (A) and ecosystem respiration (R_eco_) (B) of the *P. sylvestris* ecosystem for the years 2019–2023.
**Fig. S3.** Relationship of monthly enhanced vegetation index (EVI) and monthly gross primary productivity (GPP) (A), net ecosystem carbon exchange (NEE) (B) and ecosystem respiration (R_eco_) (C) of the *P. sylvestris* ecosystem for the years 2019–2023 assessed by linear regressions (*lm*).
**Fig. S4.** Relationship of monthly difference in net ecosystem exchange (NEE) of 2021 (Δ_NEE2021_) to the years 2019, 2020, 2022, 2023 and monthly mean air temperature (*T*_*air*_) (A), monthly maximum vapour pressure deficit (VPD_max_) (B), monthly precipitation sum (C) and monthly mean soil volumetric water content (VWC) (D). The relationship of NEE with *T*_*air*_, VPD_max_ and VWC was assessed with piecewise two segment linear regressions with the function *segmented* (*segmented*, Fasola *et al*. 2018).
**Fig. S5.** Monthly difference in net ecosystem exchange (NEE) of 2021 (Δ_NEE2021_) to the years 2020, 2022, 2023, monthly mean air temperature (*T*_*air*_) and monthly mean volumetric soil water content. Larger circles indicate more positive Δ_NEE2021_. Negative Δ_NEE2021_ values are bordered in blue, positive Δ_NEE2021_ bordered in red. Months are colour‐coded from 1 (January) to 12 (December).

## Data Availability

The data that support the findings of this study are available from the corresponding author upon reasonable request. The meteorological data and the flux data can be found on the ICOS data portal (https://www.icos‐cp.eu/data‐products/ecosystem‐release, site ID DE‐Har). Phenocam (e.g. Plapp *et al*. 2024a) and long‐term meteorological data from DE‐Har (Christen *et al*. 2024a) are available at https://zenodo.org/communities/de‐har/ in the community *Hartheim forest research site*. Time series of Phenocam pictures from both cameras can be accessed at https://phenocam.nau.edu/webcam/sites/hartheim1/ (29.6 m above ground, *hartheim1*) and https://phenocam.nau.edu/webcam/sites/hartheim2/ (8.4 m above ground, *hartheim2*).
