# Supplementary material for: Recurrent hot droughts cause persistent legacy effects in a temperate Scots Pine forest
Source: Plant Biol (Stuttg). 2025 Jun 16;28(3):622–36. doi: 10.1111/plb.70066 (PMC13089586; doi:10.1111/plb.70066)
Supplement: Supplementary file 1 — Fig. S1. Illustration of the precipitation and eddy covariance measurement positions at the two towers at DE‐Har. Photo: University of Freiburg. Fig. S2. Monthly gross primary productivity (GPP) (A) and ecosystem respiration (Reco) (B) of the P. sylvestris ecosystem for the years 2019–2023. Fig. S3. Relationship of monthly enhanced vegetation index (EVI) and monthly gross primary productivity (GPP) (A), net ecosystem carbon exchange (NEE) (B) and ecosystem respiration (Reco) (C) of the P. sylvestris ecosystem for the years 2019–2023 assessed by linear regressions (lm). Fig. S4. Relationship of monthly difference in net ecosystem exchange (NEE) of 2021 (ΔNEE2021) to the years 2019, 2020, 2022, 2023 and monthly mean air temperature (T air ) (A), monthly maximum vapour pressure deficit (VPDmax) (B), monthly precipitation sum (C) and monthly mean soil volumetric water content (VWC) (D). The relationship of NEE with T air , VPDmax and VWC was assessed with piecewise two segment linear regressions with the function segmented (segmented, Fasola et al. 2018). Fig. S5. Monthly difference in net ecosystem exchange (NEE) of 2021 (ΔNEE2021) to the years 2020, 2022, 2023, monthly mean air temperature (T air ) and monthly mean volumetric soil water content. Larger circles indicate more positive ΔNEE2021. Negative ΔNEE2021 values are bordered in blue, positive ΔNEE2021 bordered in red. Months are colour‐coded from 1 (January) to 12 (December). [file PLB-28-622-s001.docx]

**Supporting Information**

**Recurrent hot droughts cause persistent legacy effects in a temperate Scots Pine forest**

***Simon Haberstroh^1^, Andreas Christen^2^, Markus Sulzer^2^, Fabio Scarpa^1^, Christiane Werner^1^***

^1^Ecosystem Physiology, Faculty of Environment and Natural Resources, University Freiburg, Freiburg, Germany

^2^Environmental Meteorology, Faculty of Environment and Natural Resources, University Freiburg, Freiburg, Germany

*corresponding author: simon.haberstroh@cep.uni-freiburg.de, +49 761 203 8303, ORCID: 0000-0002-6097-6633

**Keywords**

Enhanced vegetation index, net ecosystem carbon exchange, sap flow, water potential,
*Pinus sylvestris, Carpinus betulus*

*
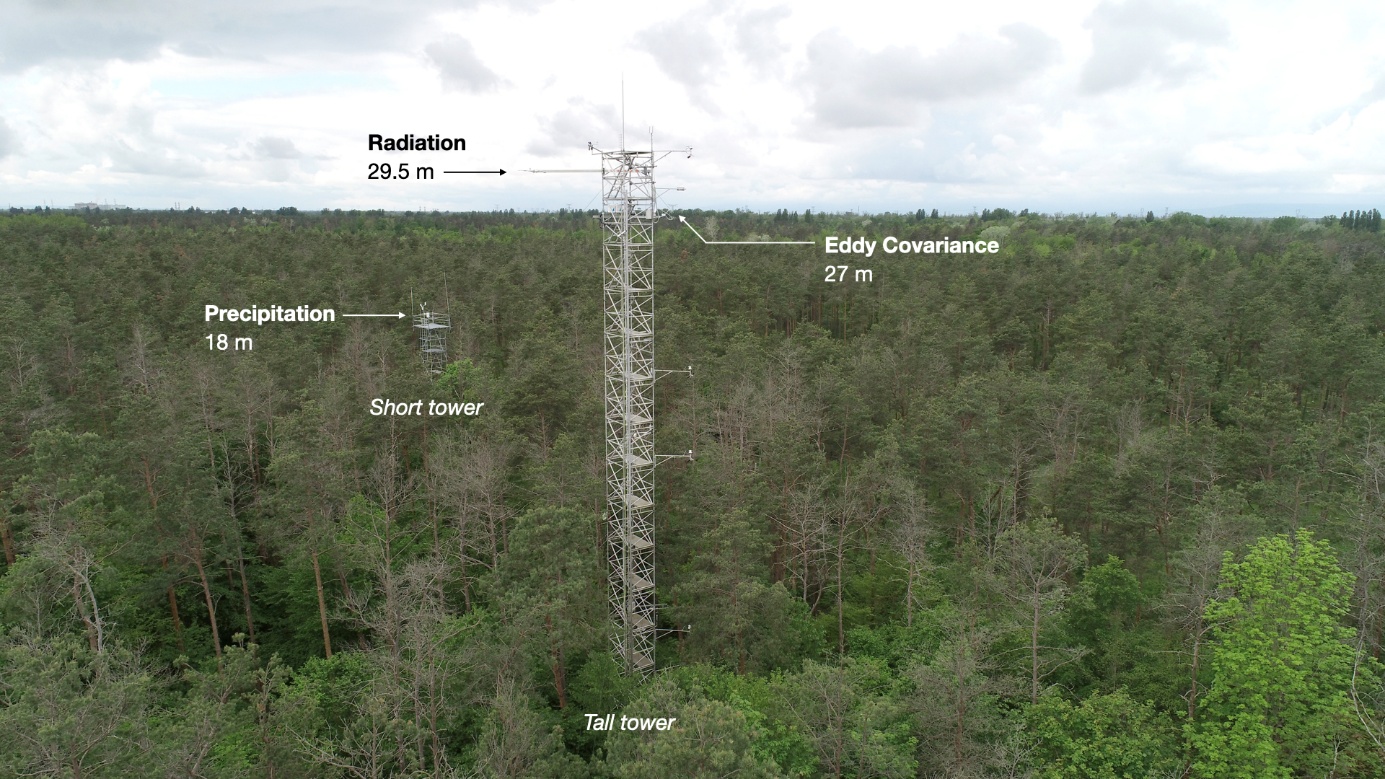
*

**Figure S1.** Illustration of the precipitation and eddy covariance measurement positions at the two towers at DE-Har. Photo: University of Freiburg.

**Methods S1 – Calculation of the enhanced vegetation index (EVI)**

The enhanced vegetation index used in this study was downloaded from the Moderate-resolution Imaging Spectroradiometer (MODIS; MOD13Q1 V6.1, Didan, 2021). According to the documentation (Didan et al., 2015), EVI was calculated as in **Equation S1**:

$EVI=G \frac{NIR-Red}{NIR+C1Red-C2Blue+L}$ (S1)

In Equation S1, the variables NIR, Red and Blue represent surface reflectances that have undergone full or partial correction for atmospheric influences. G (2.5) denotes a scaling or gain factor, L (1) signifies the canopy background adjustment, and the variables C1 (6) and C2 (7.5) represent coefficients for the aerosol resistance term (Didan et al., 2015).

For situations over bright targets (such as clouds or snow), a backup algorithm (EVI2) was applied as in **Equation S2**, to avoid unrealistically high EVI values. This algorithm does only use the red band instead of the red and blue bands:

$EVI2=2.5 \frac{NIR-Red}{NIR+2.4 Red+1}$ (S2)

For more information, please refer to Didan et al. (2015) and Didan (2021).

Didan K., Barreto Munoz, A., Solano, R., Huete, A. (2015) MODIS vegetation index user’s guide (MOD13 Series). Version 3.00, June 2015 (Collection 6). Vegetation Index and Phenology Lab, The University of Arizona. Accessed 2025-05-08 from <https://lpdaac.usgs.gov/documents/103/MOD13_User_Guide_V6.pdf>

Didan K. (2021) MODIS/Terra Vegetation Indices 16-Day L3 Global 250m SIN Grid V061 [Data set]. NASA EOSDIS Land Processes Distributed Active Archive Center. Accessed 2024-08-08 from <https://doi.org/10.5067/MODIS/MOD13Q1.061>

*

*

**Figure S2.** Monthly gross primary productivity (GPP) (A) and ecosystem respiration (R_eco_) (B) of the *P. sylvestris* ecosystem for the years 2019-2023.





**Figure S3.** Relationship of monthly enhanced vegetation index (EVI) and monthly gross primary productivity (GPP) (A), net ecosystem carbon exchange (NEE) (B) and ecosystem respiration (R_eco_) (C) of the *P. sylvestris* ecosystem for the years 2019-2023 assessed by linear regressions (*lm*).





**Figure S4.** Relationship of monthly difference in net ecosystem exchange (NEE) of 2021 (Δ_NEE2021_) to the years 2019, 2020, 2022, 2023 and monthly mean air temperature (*T_air_*) (A), monthly maximum vapour pressure deficit (VPD_max_) (B), monthly precipitation sum (C) and monthly mean soil volumetric water content (VWC) (D). The relationship of NEE with *T_air_,* VPD_max_ and VWC was assessed with piecewise two segment linear regressions with the function *segmented* (*segmented,* Fasola et al., 2018).

Fasola S., Muggeo V.M.R., Kuchenhoff K. (2018) A heuristic, iterative algorithm for change-point detection in abrupt change models. *Computational Statistics*, **33**, 997-1015.

**
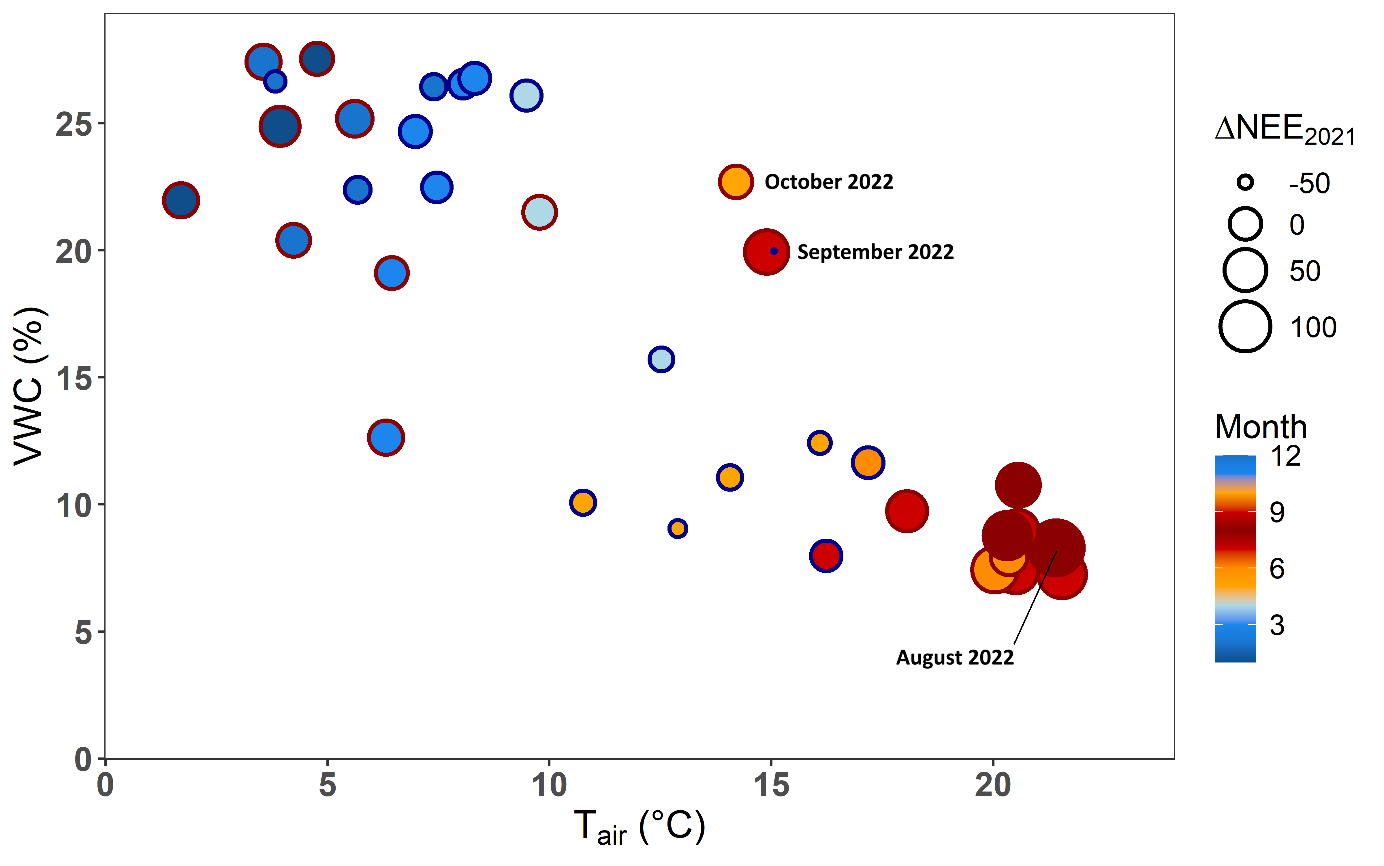
Figure S5.** Monthly difference in net ecosystem exchange (NEE) of 2021 (Δ_NEE2021_) to the years 2020, 2022, 2023, monthly mean air temperature (*T_air_*) and monthly mean volumetric soil water content. Larger circles indicate more positive Δ_NEE2021_. Negative Δ_NEE2021_ values are bordered in blue, positive Δ_NEE2021_ by red colours. Months are colour-coded from 1 (January) to 12 (December).
